# Challenges of Elderly Caregiving in the Indian Subcontinent: A Scoping Review

**DOI:** 10.1002/puh2.70085

**Published:** 2025-08-18

**Authors:** Mohammad Ishtiaque Rahman, Jahangir Alam, Forhan Bin Emdad

**Affiliations:** ^1^ Department of Computer Information Systems Thomas More University Crestview Hills Kentucky USA; ^2^ Department of Management Information Systems University of Dhaka Dhaka Bangladesh; ^3^ School of Information Florida State University Tallahassee Florida USA

**Keywords:** access disparities, caregiver burden, elderly healthcare, financial barriers, Indian subcontinent, sociocultural factors

## Abstract

This scoping review identifies the challenges in elderly caregiving across the Indian subcontinent by analyzing 21 studies selected from an initial pool of 5349 records. Key issues identified include limited access to healthcare services, high rates of multimorbidity, significant caregiver burden, and difficulties in adopting new health technologies. Additionally, the review highlights the inadequacies in healthcare infrastructure, the impact of socioeconomic factors, and financial barriers to the healthcare of the elderly in the Indian subcontinent. Urban–rural disparities, cultural norms, and the high prevalence of both chronic and infectious diseases also complicate caregiving. The study calls for comprehensive strategies to improve healthcare infrastructure, enhance financial support, strengthen government policies, and promote caregiver training and technology adoption. Addressing these challenges is crucial for improving the quality of life and healthcare for the elderly in the Indian subcontinent.

## 1 Introduction

Throughout history and now the Indian subcontinent has been one of the most populated regions globally, with about 20%–25% of the world's people at all times [1]. Like the rest of the world, Indian subcontinent population is experiencing longer lifespans. In India, the elderly population, 60 and above, will reach 300 million (24% of the total population) by 205 [2]. Bangladesh and Pakistan also face significant demographic shifts with projected elderly populations of 33 million (18% of the population) and 52 million (20% of the population), respectively [3, 4].

Elderly care in the subcontinent combines traditional family support in rural areas with emerging formal services in urban centers. Family caregiving is a fundamental aspect of elderly support throughout the region. In India, majority of elderly care is provided by family members, particularly women [5, 6]. In Bangladesh, around 80% of the elderly depend on familial support for their care. In Pakistan, family caregiving is prevalent too, with formal services having a limited role [7]. Relying on family support highlights the importance of reinforcing family and community care networks. Yet, challenges emerge due to the increasing prevalence of nuclear families and affordability issues for formal services [8, 9]. Government policies vary, with some offering modest support, whereas others face resource shortages [10].

Economic struggles, that is, poverty, worsen challenges for the elderly. In 2015, a study by the Agewell Foundation showed that more than 60% of older adults in India who are poor said they experienced neglect [11]. A similar scenario can be found in both Bangladesh and Pakistan. Elderly populations in Bangladesh and Pakistan have a hard time getting help because support services are not easily available [12, 13]. In 2018, a report from the United Nations Population Fund (UNFPA) said that 70% of old people in Bangladesh live in rural places and have limited access to needed support services [14].

The challenges of elderly caregiving are not confined to the Indian subcontinent but reflect broader trends in aging populations worldwide. Many low‐ and middle‐income countries (LMICs) face similar issues, including inadequate healthcare infrastructure, financial constraints, and caregiver burden. Countries in Latin America (e.g., Brazil and Mexico), Africa (e.g., Nigeria and South Africa), and Southeast Asia (e.g., Indonesia and the Philippines) experience comparable difficulties in ensuring adequate elderly care. A growing body of research highlights that in many LMICs, informal caregiving is the primary mode of support for the elderly, often placing an unsustainable burden on families, particularly women. Given the global rise in aging populations and the increasing demand for caregiving support, the insights from this study are not only relevant to South Asia but also provide a comparative foundation for addressing similar challenges in other regions. By understanding the structural and socioeconomic barriers present in elderly caregiving across the Indian subcontinent, policymakers and researchers in other LMICs can develop informed strategies to improve elderly care on a broader scale.

Previous studies have identified various challenges in elderly caregiving in the Indian subcontinent, including poverty, loneliness, a shortage of caregivers, and limited access to primary care facilities, as reasons for the lack of quality care they receive. However, the combination of limited resources and a large population makes it challenging to conduct a single, comprehensive study to identify all the challenges associated with elderly care in this region. Fortunately, the socioeconomic environment in the Indian subcontinent has many common characteristics. Challenges identified in one region of one country can be easily found in other regions without many differences. To address this, it is essential to understand the challenges identified by all relevant research in this area. This will help in building effective care systems, developing policies focused on care, and creating societies that respect and support our elders. Unlike previous studies that examine elderly caregiving challenges in isolated national contexts, this review systematically consolidates findings from Bangladesh, India, and Pakistan, offering a comparative perspective on caregiving across the Indian subcontinent. This synthesis highlights regional commonalities as well as unique country‐specific challenges, providing a more nuanced understanding of caregiving disparities. Moreover, this review goes beyond existing research by examining the intersection of sociocultural norms, economic constraints, and healthcare infrastructure multidimensional analysis that has not been comprehensively explored before. By identifying emerging challenges such as the digital divide in elderly care, increasing caregiver burden due to shifting family structures, and disparities in policy implementation, this study offers a deeper insight into how systemic issues impact caregiving in South Asia. So, the goal of this study is to identify, consolidate, and generalize the existing challenges in elderly care in the countries of the Indian subcontinent.

## 2 Methods

The objective of this article is to understand the challenges of elderly caregiving in the Indian subcontinent. To achieve this objective, a scoping literature review was conducted. A scoping review is an appropriate approach for this study as it allows for the synthesis of evidence from multiple sources, providing a comprehensive overview of the challenges faced in elderly caregiving across the Indian subcontinent [15]. This approach ensures that the findings are representative and robust and can be used to inform policy and practice.

**FIGURE 1:**
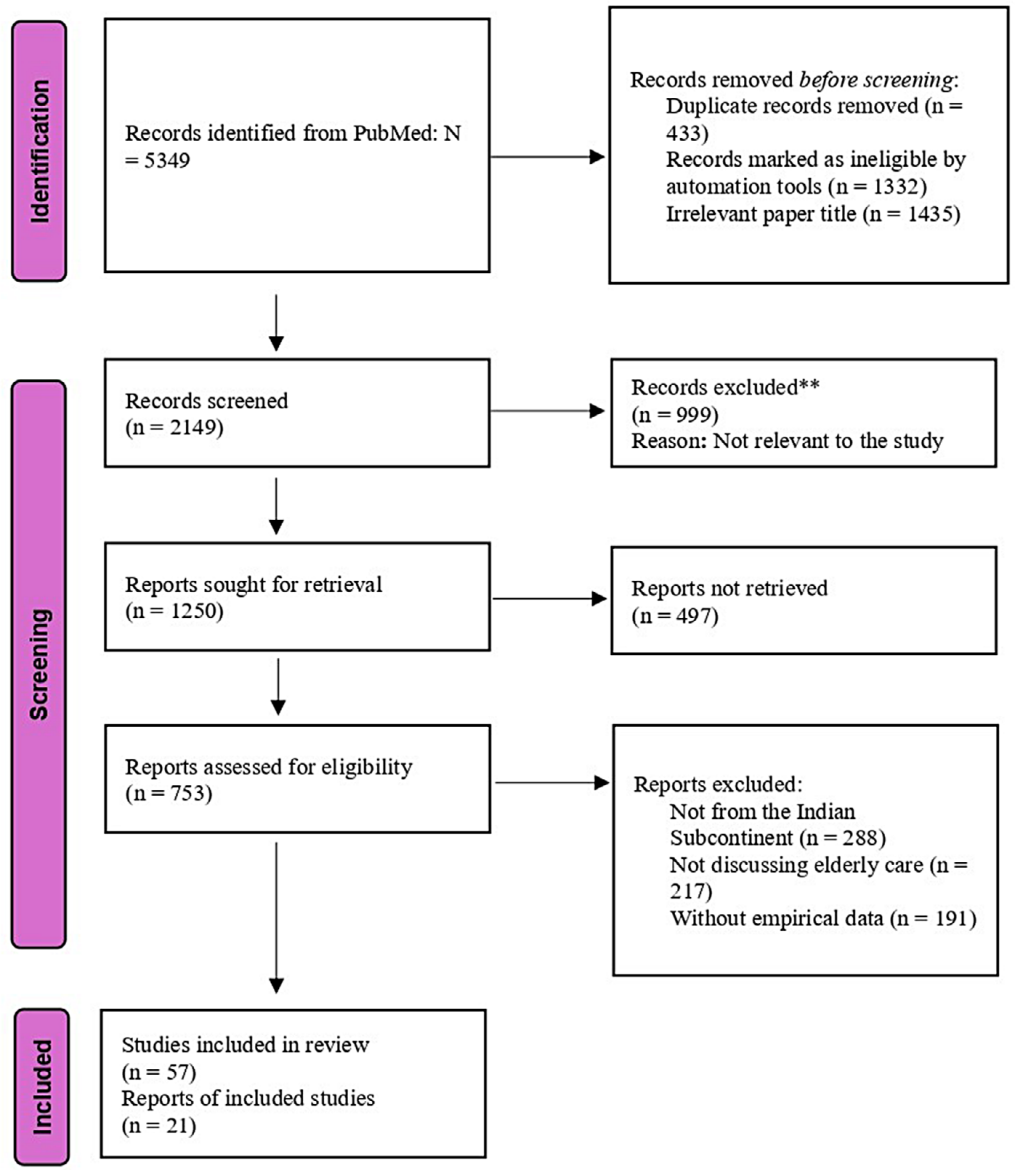
PRISMA diagram.

The PRISMA‐ScR (Preferred Reporting Items for Systematic Reviews and Meta‐Analyses extension for Scoping Reviews) model was used as a framework for identifying, screening, and selecting the studies for review (Figure 1) [16]. A comprehensive search was conducted in PubMed, a primary database for biomedical and health sciences literature, to identify relevant studies. The following search terms were used:

(elderly OR geriatric OR older OR senior OR aging OR palliative) AND (care OR caregiving OR healthcare) AND (challenges OR issues OR problems) AND “Country”

This search strategy was designed to retrieve a wide range of studies discussing elderly caregiving challenges across India, Bangladesh, and Pakistan.

### 2.1 Eligibility Criteria

*Inclusion Criteria:*

– Studies focused on elderly caregiving challenges in India, Bangladesh, or Pakistan.
– Published in English.
– Empirical studies, literature reviews, and policy analyses discussing elderly care.

*Exclusion Criteria:*

– Studies that do not specifically address caregiving challenges.
– Articles covering general geriatric healthcare without a caregiving focus.
– Opinion pieces or studies without empirical data.

### 2.2 Study Selection Process

The study selection followed a three‐stage process:

1. Title and abstract screening—Initial screening to remove irrelevant studies.
2. Full‐text review—Articles meeting the inclusion criteria were assessed in full.
3. Final selection—Studies providing relevant insights into elderly caregiving challenges were included for synthesis.

The selection process is summarized in the PRISMA‐ScR Flow Diagram (Figure 1).

### 2.3 Data Extraction and Synthesis

Data were extracted using a structured approach, focusing on study characteristics such as author, year, country, sample size, and methodology. Key findings related to elderly caregiving challenges were identified and categorized into thematic areas. Emerging themes and research gaps were synthesized to highlight common patterns across literature. As scoping reviews do not require formal quality assessment, the included studies were analyzed on the basis of their relevance and contributions to the topic. This thematic synthesis approach ensures a comprehensive understanding of the various caregiving challenges, facilitating the identification of policy and practice implications within the Indian subcontinent.

## 3 Results

**FIGURE 2:**
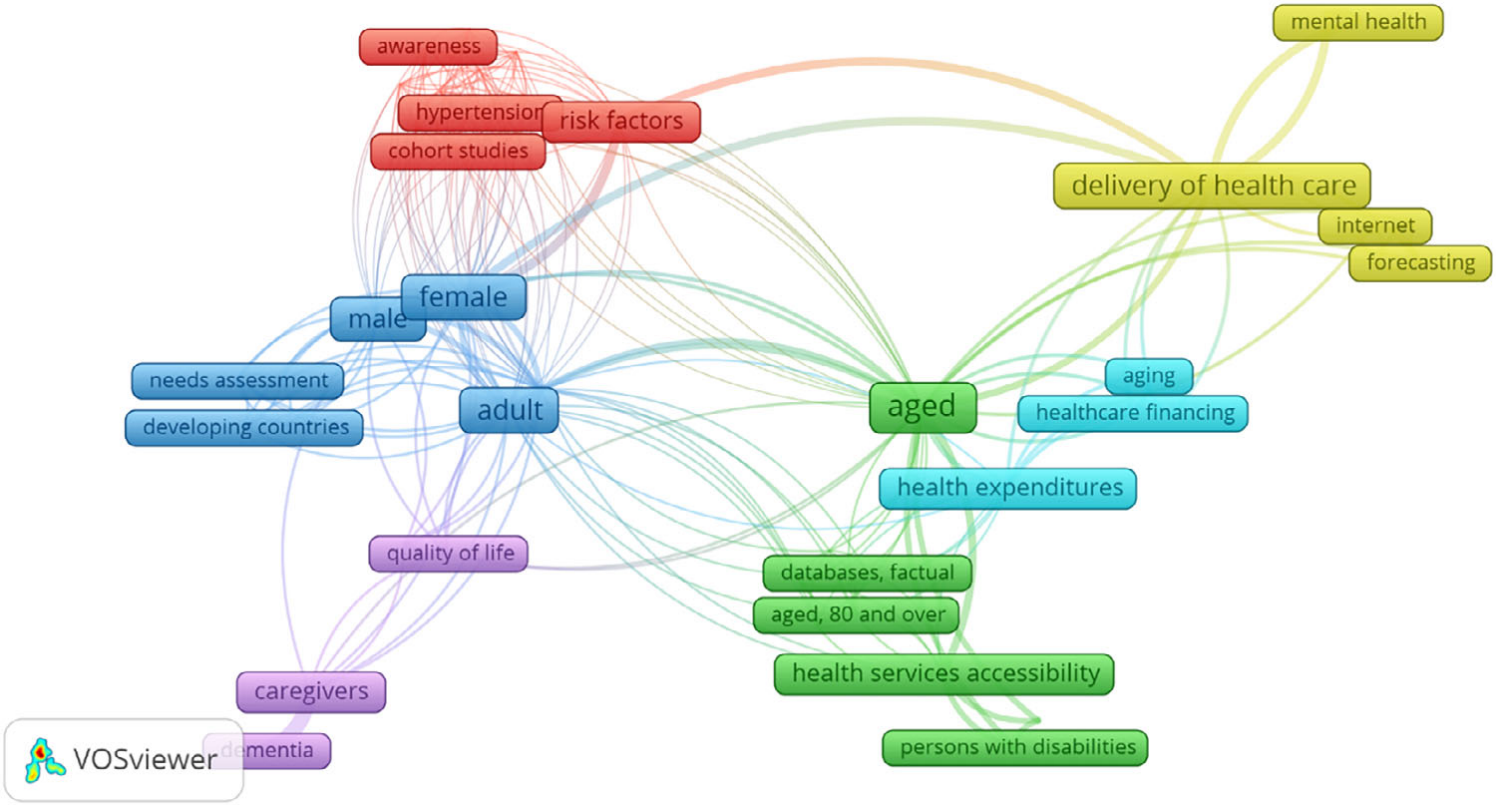
Text data‐based term co‐occurrence map.

The initial 5349 records from PubMed decreased to 2149 after the initial screening and removal of duplicates. Upon reviewing the abstracts, 999 were excluded as irrelevant to the study, and 1250 were considered for retrieval. Due to various reasons, 497 studies could not be retrieved, and 753 studies were assessed for inclusion eligibility in the study. In the end, 21 studies were found eligible for inclusion in the study (Table 1). Figure 2 presents a term co‐occurrence map of the most recurring topics. After the review, the following challenges have been identified as the most pressing in elderly caregiving in the Indian subcontinent.

**TABLE 1:** Studies included in the review.

Author	Year	Country	Sample	Method	Study focus
Hamiduzzaman et al. [13]	2022	Bangladesh	Older Bangladeshi women (*n* = 22) and Health staff (*n* = 11)	Semi‐structured, in‐depth interviews	Relationship with caregiver affects healthcare utilization and service‐seeking behavior
Jahan et al. [17]	2022	Bangladesh	Marginalized older people (*n* = 636)	Cross‐sectional, semi structured interview	High out‐of‐pocket expenses and lack of awareness influence healthcare‐seeking behavior
Hamiduzzaman [18]	2020	Bangladesh	Rural elderly women (*n* = 4930)	Mixed method	Seasonal symptoms and diseases impact primary healthcare utilization in rural areas
Chatterjee et al. [19]	2022	India	Older adults (*n* = 45,299)	Survey	Economic status, education, and residence significantly influence inpatient health expenditure
Gupta et al. [20]	2022	India	Elderly patients with respiratory diseases (*n* = 500)	Cross‐sectional	Low healthcare utilization due to lack of awareness, affordability, and accessibility
Joe et al. [21]	2015	India	Older adults (*n* = 34,831)	Nation‐wide survey	Horizontal inequity in healthcare utilization among elderly individuals in India
Agrawal et al. [22]	2014	India	Older widows (*n* = 10,111)	National Sample Survey	Economic and social factors affect health‐seeking behavior and morbidity patterns
Puri et al. [23]	2022	India	Aboriginal older adults (*n* = 11,365)	Longitudinal Ageing Study	Multimorbidity is linked to increased healthcare utilization and expenditure
Sara et al. [24]	2018	Bangladesh	Older adults (*n* = 566)	Cross‐sectional	High prevalence of multimorbidity among the elderly population
Sinha et al. [25]	2022	India	Older adults (*n* = 59,073)	Longitudinal Ageing Study, observational analysis	Social determinants impact diabesity and its association with multimorbidity
Chauhan et al. [26]	2022	India	Older adults (*n* = 31,373)	Nationally representative survey	Prevalence and factors associated with chronic disease multimorbidity
Chauhan et al. [27]	2022	India	Older adults (*n* = 72,250)	Nationally representative survey	Noncommunicable diseases‐related inequalities among older adults
Goli et al. [28]	2014	India	Older adults (*n* = 34,831)	National Sample survey	Socioeconomic determinants of health inequalities among older population in India
Shah et al. [29]	2020	Pakistan	Caregivers of palliative care patients (*n* = 250)	Cross‐sectional	Perceptions, knowledge, and attitudes towards the concept and approach of palliative care among caregivers
Sabzwari et al. [30]	2016	Pakistan	Elderly caregivers (*n* = 350)	Cross‐sectional	Burden and associated factors for caregivers of the elderly in a developing country
Bilal et al. [31]	2020	Pakistan	Care home staff (*n* = 27)	Semi‐structured interviews	Perceptions and experiences of care home staff in Pakistan during the COVID‐19 pandemic
Sarker et al. [32]	2023	Bangladesh	Older citizens (*n* = 27)	In‐depth interviews	Explored healthcare‐seeking experiences of older citizens in Bangladesh
Hoque et al. [33]	2017	Bangladesh	Elderly population (*n* = 300)	Structured questionnaire survey	Studied factors influencing the adoption of mHealth by the elderly in Bangladesh
Kabir et al. [34]	2003	Bangladesh	Older people in rural and urban areas (*n* = 786)	Descriptive multi‐dimensional survey	Examined gender and rural–urban differences in reported health status by older people in Bangladesh
Naz et al. [35]	2021	Pakistan	Older adults (*n* = 5319)	Cross‐sectional, nationally survey	Investigated behavioral factors associated with the utilization of healthcare services among the elderly in Pakistan
Qidwai et al. [36]	2009	Pakistan	Geriatric patients (*n* = NA)	Editorial	Described geriatric patients’ expectations of their physicians in a tertiary care hospital in Pakistan

### 3.1 Elderly Caregiving Challenges

#### 3.1.1 Limited Access to Healthcare Services

Elderly individuals in Bangladesh, India, and Pakistan frequently encounter limited access to healthcare services due to issues such as socioeconomic status, rural–urban distinctions, and cultural barriers [27, 35]. Research by Jahan et al. in Bangladesh highlighted that older individuals living in marginalized urban slums face limited healthcare access due to limited awareness, inadequate services, and financial constraints [17]. In India, studies by Chauhan et al. revealed that older adults with multiple chronic conditions encounter significant inequalities in healthcare access [26]. Additionally, Joe et al. reported horizontal inequities limiting healthcare access among the elderly in India [21]. In Pakistan, Naz et al. found that behavioral factors, such as a lack of awareness and adherence to traditional beliefs, further limit healthcare access among the elderly population [27, 35].

#### 3.1.2 Multimorbidity and Healthcare Utilization

Multimorbidity is common among the elderly in Bangladesh, India, and Pakistan, causing higher healthcare usage and putting additional pressure on the healthcare systems [23, 24, 25]. In Bangladesh, Sara et al. found a high occurrence of multimorbidity among the elderly, influencing the use of healthcare services [24]. Sinha et al. discovered that diabesity's presence and its connection to multimorbidity among older adults led to heightened healthcare need in India [25]. Puri and Pati similarly found that older adults in India with noncommunicable disease (NCD) multimorbidity tend to require healthcare services more frequently [23].

#### 3.1.3 Caregiver Burden

Caregivers of the elderly population in the Indian subcontinent face considerable challenges, that is, insufficient knowledge, training, and resources, leading to increased emotional, physical, and financial burdens on caregivers [28, 29]. Goli et al. discovered that socioeconomic factors contribute to health disparities among the elderly, putting a greater strain on caregivers, particularly those with lower socioeconomic status [28]. Similarly, Shah et al. reported that in Pakistan, older patients have high expectations from their caregivers, contributing to caregiver burden [29].

#### 3.1.4 New Technologies Adoption Challenges for Elderly Care

The adoption of new technologies, like mHealth, has the potential to enhance elderly care in the subcontinent, but challenges in implementation arise from factors such as the digital divide, lack of awareness, and resistance to change [19, 33]. In Bangladesh, Hoque and Sorwar identified that factors influencing the adoption of mHealth among caregivers include perceived usefulness, social influence, and facilitating conditions [33]. Chatterjee et al. reported that adopting digital health technologies in India can improve healthcare access for older adults but requires overcoming challenges related to affordability, accessibility, and awareness [19].

#### 3.1.5 Inadequate Healthcare Infrastructure and Human Resources

In Bangladesh, India, and Pakistan, the healthcare infrastructure and workforce are often insufficient to meet the needs of the growing elderly population. Inadequate facilities, lack of specialized geriatric care, and insufficient numbers of trained healthcare professionals contribute to the challenges faced in providing elderly care in these countries [19, 33, 36]. Hoque and Sorwar and Sarker et al. reported that Bangladesh's healthcare system is plagued by inadequate infrastructure and a lack of healthcare professionals trained in geriatric care [33]. In India, Chatterjee et al. found that the healthcare system faces challenges in meeting the needs of the elderly population due to insufficient geriatric care services and a lack of trained healthcare professionals [19]. Similarly, in Pakistan, Qidwai et al. reported that the healthcare system struggles to cater to the needs of the elderly population due to inadequate healthcare infrastructure, limited geriatric care services, and a shortage of trained healthcare professionals [36].

#### 3.1.6 Social Determinants of Health

Social determinants, such as socioeconomic status, education, and social support, play a significant role in the health outcomes of older adults in Bangladesh, India, and Pakistan [28, 30, 34]. In Bangladesh, Kabir et al. found that socioeconomic factors, including poverty, illiteracy, and lack of social support, contribute to the poor health status of the elderly population [34]. In India, Goli et al. and Agrawal et al. reported that socioeconomic factors contribute to health inequalities among the elderly, with those from lower socioeconomic backgrounds experiencing poorer health outcomes [22, 28]. Similarly, in Pakistan, Sabzwari et al. found that socioeconomic factors, such as education and income, impact the health and well‐being of older adults [30]. *Lack of healthcare financing support*: In Bangladesh, healthcare financing relies heavily on out‐of‐pocket payments, which can lead to financial hardship for older adults and their families [34]. In India, although the government has initiated health insurance schemes for the elderly population, the coverage remains inadequate and inconsistent [19]. Gupta et al. identified that one of the main causes of low healthcare utilization of older adults was lack of awareness, affordability, and accessibility [20]. In Pakistan, healthcare financing is a mixture of public and private sectors, but older adults often face financial barriers to accessing healthcare services due to limited public funding and high out‐of‐pocket expenses [35].

#### 3.1.7 Lack of Government Policy and Programs

In Bangladesh, there is a lack of comprehensive national policies and programs addressing the needs of the elderly population [17]. In contrast, India has developed the National Program for the Health Care of the Elderly (NPHCE), which aims to provide preventive, curative, and rehabilitative services for older adults [11]. However, the implementation of this program is still limited in scope. In Pakistan, the government has also made efforts to establish programs for the elderly population, such as the Pakistan Bait‐ul‐Mal program, which offers financial support for healthcare expenses, but its effectiveness is limited due to resource constraints [35].

#### 3.1.8 Cultural Differences and Caregiver Perceptions

In Bangladesh, societal expectations and cultural norms dictate that family members, especially women, should provide care for older adults [37]. This may lead to caregiver burden and stress. In India, societal changes and the emergence of nuclear families have led to a decline in the traditional caregiving roles within families [28]. This has resulted in an increased demand for formal elderly care services. In Pakistan, traditional family structures are still prevalent, and older adults often rely on their families for care [30]. However, caregiver burden and the need for formal support services are also emerging concerns in Pakistan [31, 36].

#### 3.1.9 Urban–Rural Disparities

In Bangladesh, rural elders face inadequate healthcare infrastructure, a shortage of trained geriatric professionals, and reliance on traditional medicine due to limited formal healthcare access. Transportation barriers further delay diagnoses and treatment, worsening health outcomes [18]. In India, rural areas struggle with poor healthcare accessibility, fewer medical professionals, and underdeveloped geriatric services, forcing elders to travel long distances for specialized care. Although urban areas have better hospitals, affordability is a major challenge, especially for lower‐income groups. State‐sponsored programs like Ayushman Bharat aim to address disparities, but coverage remains inconsistent [19]. In Pakistan, both urban and rural areas lack sufficient geriatric healthcare infrastructure. Rural elders rely on home remedies or unqualified practitioners, whereas urban elders face financial constraints despite better facilities. Inadequate policy implementation results in delayed medical interventions and a higher disease burden [35].

#### 3.1.10 Disease Prevalence and Management

In Bangladesh, NCDs such as hypertension, diabetes, cardiovascular diseases, and arthritis are prevalent among the elderly population, requiring long‐term care and management. However, the lack of preventive healthcare services and geriatric‐specific medical programs limits proper disease management, often leading to severe complications. Many elderly individuals, particularly those in rural areas, delay seeking medical care due to financial constraints and limited access to healthcare facilities, further exacerbating their health conditions. Mental health issues such as depression and anxiety are also rising concerns, yet they remain largely unaddressed due to societal stigma and inadequate mental healthcare services [18]. In India, the burden of NCDs is similarly high among older adults, with diabetes, cardiovascular diseases, and chronic respiratory conditions being the most prevalent. However, the persistence of communicable diseases like tuberculosis remains a significant challenge, especially among elderly individuals living in low‐income and rural areas. The coexistence of both communicable and NCDs creates a dual burden on healthcare services, making it difficult for elderly individuals to receive integrated care. Many elderly patients face challenges in medication adherence, regular monitoring, and access to specialized geriatric services, increasing their risk of severe complications. Government health initiatives such as the NPHCE aim to address these issues, but implementation remains uneven, particularly in rural areas [19]. In Pakistan, the elderly population faces a high burden of NCDs, including hypertension, diabetes, and cardiovascular diseases, like Bangladesh and India. However, Pakistan also has a high prevalence of mental health issues and geriatric syndromes, such as falls, frailty, and cognitive decline, which require specialized care and management. Unfortunately, mental health services are severely lacking, and geriatric mental health remains an underdeveloped area of healthcare policy. Many elderly individuals do not seek help with mental health conditions due to cultural stigmas and a lack of awareness. Additionally, limited availability of long‐term care facilities means that elderly patients with severe disabilities or chronic conditions often rely entirely on family caregivers, further increasing the burden on household resources. There is an urgent need for specialized geriatric care programs, better integration of mental health services, and greater investments in chronic disease management initiatives to address the rising healthcare needs of Pakistan's aging population [30, 35].

### 3.2 Intra‐Country Variations in Elderly Care Across South Asia

Elderly care in South Asia exhibits significant intra‐country disparities, influenced by factors such as economic conditions, healthcare infrastructure, policy implementation, and sociocultural dynamics. Recognizing these regional differences is essential for developing targeted policies that address the unique needs of elderly populations in various states, provinces, and districts.

#### 3.2.1 India: Regional Inequalities in Elderly Care

India's diverse landscape results in notable disparities in geriatric healthcare across its states. Southern states like Kerala have made considerable progress in elderly care, whereas northern and eastern states such as Bihar face ongoing challenges. Kerala boasts the highest proportion of elderly individuals in India, with 16.5% of its population aged 60 and above. The state has invested in elderly friendly healthcare services, including specialized geriatric wards in district hospitals and community‐based palliative care programs. Despite these advancements, Kerala reports a high prevalence of multimorbidity among older adults, with 59.2% experiencing multiple chronic conditions [38]. This paradox may be attributed to better health awareness and reporting, as well as a higher life expectancy leading to age‐related health issues.

In contrast, Bihar has the lowest proportion of elderly residents, at 7.7%. The state faces significant challenges in providing adequate geriatric care, as public healthcare utilization by the elderly is notably low, with only 9.7% accessing public health facilities. Additionally, a study comparing health‐related expenditures in Bihar and Kerala found that only 8.22% of the elderly in Bihar had some form of insurance coverage, leading to higher out‐of‐pocket expenses [39].

Urban–rural disparities further exacerbate these challenges. In Maharashtra, for instance, urban centers like Mumbai offer well‐established public and private hospitals with geriatric services, whereas rural districts such as Gadchiroli lack basic healthcare infrastructure, compelling elderly individuals to travel long distances for medical attention [26]. This urban–rural divide highlights the need for region‐specific healthcare strategies. These regional disparities call for localized elderly care policies, ensuring that underdeveloped states receive more funding for geriatric healthcare, rural regions gain better access to primary care, and insurance coverage expands to reduce financial burdens on the elderly population [38].

#### 3.2.2 Bangladesh: Urban–Rural Disparities in Elderly Care

In Bangladesh, elderly care challenges are pronounced between urban centers and rural areas. Major cities like Dhaka and Chittagong have public and private hospitals equipped with geriatric care units. However, high out‐of‐pocket payments pose significant barriers, especially for low‐income elderly individuals. A study highlighted that although healthcare facilities are available, the associated costs, lack of caregivers, and travel distances hinder service utilization among older citizens [40].

Rural regions, including Barisal and Sylhet, face more severe challenges. Healthcare facilities in these areas are sparse, and specialized geriatric services are almost nonexistent. Many elderly individuals must travel long distances to receive medical care, which is both physically and financially burdensome. The lack of trained geriatric professionals further exacerbates the situation, with many elderly patients relying on community health workers or informal caregivers who may not have the necessary expertise [41]. The Old Age Allowance Scheme, which provides financial assistance to elderly individuals, is better implemented in urban areas, where beneficiaries are more aware of how to navigate the bureaucratic system. In contrast, many rural elderly individuals are either unaware of the scheme or unable to access it due to administrative hurdles. This results in a significant portion of the rural elderly population remaining without any formal financial or healthcare support [42].

#### 3.2.3 Pakistan: Provincial Differences in Elderly Care Infrastructure

Pakistan exhibits significant disparities in elderly healthcare access across its provinces, with Punjab and Sindh possessing relatively better healthcare infrastructure, whereas Balochistan and Khyber Pakhtunkhwa (KPK) face severe shortages of medical professionals and facilities. In Punjab, as the most developed province, cities like Lahore and Rawalpindi offer specialized geriatric departments and outpatient services. The Sehat Sahulat Program (SSP), initiated in KPK in 2015 and later expanded to Punjab, provides free healthcare to low‐income individuals, enhancing access to necessary medical treatments for the elderly. The program's success in Punjab is attributed to better infrastructure and resource allocation [43].

Conversely, Balochistan faces significant challenges in elderly healthcare delivery. The province has the lowest gains in life expectancy, with minimal improvements observed between 1990 and 2019 [44]. Large rural areas lack hospitals with geriatric care facilities, compelling elderly individuals to travel long distances to cities like Quetta or rely on unqualified healthcare providers. The financial burden on the elderly in this province is exceptionally high, exacerbated by limited social security schemes.

Similarly, in KPK's mountainous regions, geographic isolation severely limits access to healthcare. Although the SSP was first introduced in KPK, challenges persist due to infrastructure limitations and low internet penetration, hindering the development of telemedicine solutions [43]. In Sindh, particularly Karachi, the healthcare landscape is mixed, with elderly care services largely privatized. Those who can afford private hospitals receive high‐quality care, whereas low‐income elderly individuals contend with overburdened public hospitals and high out‐of‐pocket expenses. Rural Sindh mirrors Balochistan's situation, with many districts lacking basic healthcare infrastructure, severely limiting access to specialized elderly care [45]. These provincial disparities underscore the need for targeted policies and resource allocation to address the unique challenges faced by the elderly population in each region.

## 4 Discussion

**FIGURE 3:**
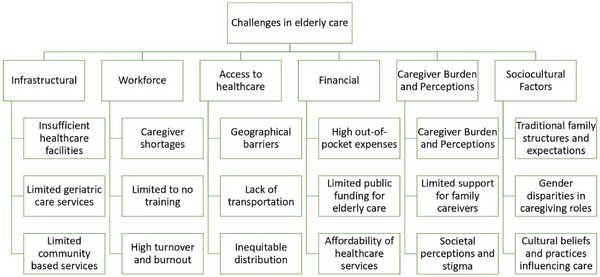
Taxonomy of elderly caregiving challenges in Indian subcontinent.

Although previous research has identified various barriers to elderly caregiving in South Asia, this study uniquely synthesizes findings across multiple national contexts to reveal broader regional trends and gaps in policy responses. One of the key contributions of this review is its identification of emerging, understudied challenges such as the growing reliance on informal caregiving networks due to urbanization, the slow adaptation of technology‐assisted care models, and disparities in caregiver support services across rural and urban areas. This review also sheds light on how traditional family caregiving models are evolving due to economic pressures and demographic transitions, an aspect that has been largely overlooked in prior studies. Additionally, by situating these challenges within an international caregiving framework, the study establishes comparative relevance with other LMICs facing similar demographic and healthcare transformations. The challenges identified in providing elderly healthcare in Indian subcontinent can be categorized into six main themes (Figure 3): infrastructural, workforce, access to healthcare, financial, caregiver burden and perceptions, and sociocultural factors.

### 4.1 Financial Burden and Healthcare Access

A significant challenge across all three countries is the high financial burden on older adults, with out‐of‐pocket expenses creating barriers to healthcare access [19, 34, 35, 46]. This financial strain often results in inequitable access and poorer health outcomes among elderly populations. Expanding public financing mechanisms, increasing health insurance coverage, and implementing social protection schemes can mitigate these challenges. Additionally, targeted financial aid programs should be introduced to support elderly individuals in low‐income and rural communities, ensuring equitable access to essential healthcare services. Developing sustainable microinsurance models and expanding government‐funded geriatric care programs can provide long‐term financial security for elderly patients [17, 19, 34, 35]. For low‐income urban elderly populations, government‐sponsored health insurance should be expanded to cover chronic disease management and home‐based care. In rural areas, subsidized health services and free medication programs can address cost‐related access issues. In middle‐income urban areas, incentives for private healthcare providers to offer affordable geriatric care can improve service availability.

### 4.2 Caregiver Burden and Workforce Shortages

Caregivers in the Indian subcontinent face significant physical, emotional, and financial stress due to insufficient knowledge, training, and resources [29, 30, 47]. Factors such as social expectations and cultural norms further exacerbate caregiver burden, particularly among women [28]. Addressing these challenges requires structured caregiver training programs, financial incentives for family caregivers, and greater access to respite care services [29, 48, 49]. For rural caregivers, mobile training units and community caregiving workshops can provide basic geriatric care skills. For urban caregivers, formal caregiving networks should be developed to provide respite care services and emotional support groups. Middle‐class caregivers balancing work and family duties would benefit from tax relief programs and employer‐supported caregiving benefits.

### 4.3 Global Comparisons and Relevance

The findings of this study align with global research on elderly caregiving, demonstrating that caregiving challenges are not unique to South Asia. Countries in Africa, Latin America, and Southeast Asia face similar structural barriers, making this study highly relevant to international discussions on elderly care. For example, in Brazil and Mexico, families remain the primary caregivers due to the lack of comprehensive elderly care infrastructure, leading to high rates of caregiver stress—a pattern observed in India, Bangladesh, and Pakistan. Similarly, in Nigeria and South Africa, financial barriers and out‐of‐pocket expenses limit access to essential healthcare services for elderly individuals [19, 34, 35].

### 4.4 Urban–Rural Disparities and Disease Management

Studies from Indonesia and the Philippines highlight that urban–rural disparities create significant inequities in elderly care, a trend mirrored in South Asia. Just as in Bangladesh and India, rural elderly populations in Southeast Asia struggle with limited healthcare access due to inadequate infrastructure and financial constraints [18, 50]. In contrast, urban elderly populations face social isolation and affordability challenges, particularly as traditional family support structures decline [19]. For rural elderly populations, increasing mobile healthcare clinics, community‐based telemedicine programs, and incentives for medical professionals to work in remote areas can improve healthcare access. In urban areas, developing affordable geriatric‐focused housing communities with integrated health and social services can reduce isolation and financial strain.

Additionally, disease prevalence among elderly individuals in South Asia presents a dual burden of NCDs such as hypertension, diabetes, and cardiovascular diseases, alongside persistent communicable diseases like tuberculosis [18, 19, 49]. The absence of structured geriatric care services leads to delayed diagnoses and inconsistent disease management, requiring more robust chronic disease care programs and specialized caregiver education initiatives.

### 4.5 Policy Recommendations and Future Directions

The study's findings align with international policy priorities, particularly the WHO Decade of Healthy Ageing (2021–2030), which calls for integrated, person‐centered approaches to elderly care. Strengthening public–private partnerships, expanding telemedicine services, and promoting community‐based caregiving models can significantly enhance elderly care across the region. For low‐income elderly populations, targeted government subsidies and nonprofit collaborations should focus on free geriatric healthcare and community meal programs. For middle‐income populations, affordable insurance schemes should expand coverage for home‐based care and chronic disease management. For rural elderly people, localized senior‐friendly transportation services and mobile healthcare units can bridge service gaps.

To ensure equitable access, policies should focus on rural outreach programs, subsidized healthcare services for low‐income families, and workforce development for specialized geriatric care. By situating this study within an international context, the findings can inform not just regional policies but also contribute to the global discourse on aging and caregiving. This makes the study highly relevant for policymakers, researchers, and healthcare professionals developing elderly care strategies worldwide.

### 4.6 Addressing Structural Gaps in Elderly Care

The elderly care crisis in the Indian subcontinent is worsened by caregiver shortages, infrastructural deficiencies, and limited healthcare access [18, 27, 51, 52]. With a rapidly aging population, the demand for caregivers far exceeds supply, leading to inadequate support for elderly individuals [28]. Poorly maintained senior care facilities and limited geriatric training programs further constrain service availability [33, 36]. Expanding geriatric education programs, developing a structured licensing framework for caregivers, and increasing investments in age‐friendly healthcare facilities can help build a more sustainable and inclusive elderly care system [52, 53, 54].

Strategies to strengthen elderly care systems include enhancing coordination between healthcare sectors, investing in social determinants of health, and implementing preventive healthcare programs for older adults [19, 54, 55]. Additionally, leveraging public–private partnerships, community‐based care models, and digital health solutions like telemedicine can provide cost‐effective and scalable solutions for elderly care challenges in the Indian subcontinent. By implementing these strategies, policymakers can create sustainable, inclusive, and high‐quality elderly care systems that prioritize dignity, accessibility, and affordability for aging populations across South Asia.

## 5 Conclusion

This review highlights key challenges in elderly caregiving across Bangladesh, India, and Pakistan, including limited healthcare access, multimorbidity, caregiver burden, technological adoption barriers, inadequate infrastructure, and socioeconomic disparities. Financial constraints, insufficient policies, cultural norms, and urban–rural disparities further complicate elderly care. Addressing these issues requires strengthening healthcare infrastructure, financial support, government policies, caregiver training, and digital integration to enhance elderly care services in the region. These findings are relevant beyond South Asia, as many LMICs face similar caregiving challenges. The insights from this study can inform global elderly care policies by identifying effective interventions adaptable to diverse socioeconomic contexts. Addressing caregiver burden, financial hardship, and inadequate healthcare access requires collaborative international efforts. Future research should focus on cross‐country comparisons to identify the best practices in elderly caregiving. International policy collaborations must prioritize support for informal caregivers, expansion of geriatric healthcare, and sustainable long‐term care solutions. By incorporating these insights, global stakeholders can advance more inclusive and effective elderly caregiving strategies.

## Author Contributions

**Mohammad Ishtiaque Rahman**: conceptualization, investigation, writing – original draft, methodology, formal analysis. **Jahangir Alam**: writing – original draft, methodology, formal analysis, writing – review and editing. **Forhan Bin Emdad**: writing – review and editing, writing – original draft, methodology, formal analysis.

## Disclosure

The authors declare that there are no commercial, financial, personal, or professional relationships that could be construed to influence the objectivity, integrity, or value of this study.

## Ethics Statement

This literature review did not involve any new studies with human participants or animals performed by any of the authors. All data analyzed were from publicly available sources, and the review was conducted in accordance with ethical guidelines for the use of published research.

## Conflicts of Interest

The authors declare no conflicts of interest.

## Data Availability

No new data were created or analyzed in this study. Data sharing is not applicable to this article as it is based solely on a review of existing literature. The sources of data for the reviewed studies are provided in the references section.
